# Can eHealth programs for cardiac arrhythmias be scaled-up by using the KardiaMobile algorithm?

**DOI:** 10.1016/j.cvdhj.2023.11.004

**Published:** 2023-11-14

**Authors:** Bridget M.I. Slaats, Sebastiaan Blok, G. Aernout Somsen, Igor I. Tulevski, Reinoud E. Knops, Bert-Jan H. van den Born, Michiel M. Winter

**Affiliations:** ∗ Cardiology Centers of the Netherlands, Utrecht, The Netherlands; † Department of Internal Medicine, Amsterdam Cardiovascular Sciences, Amsterdam UMC, Amsterdam, The Netherlands; ‡ Department of Cardiology, Amsterdam UMC, Amsterdam, The Netherlands

**Keywords:** Atrial fibrillation, Diagnostic accuracy, HartWacht program, Single-lead electrocardiography, Sinus rhythm

## Abstract

**Background:**

Remote monitoring devices for atrial fibrillation are known to positively contribute to the diagnostic process and therapy compliance. However, automatic algorithms within devices show varying sensitivity and specificity, so manual double-checking of electrocardiographic (ECG) recordings remains necessary.

**Objective:**

The purpose of this study was to investigate the validity of the KardiaMobile algorithm within the Dutch telemonitoring program (HartWacht).

**Methods:**

This retrospective study determined the diagnostic accuracy of the algorithm using assessments by a telemonitoring team as reference. The sensitivity, specificity, negative predictive value (NPV), positive predictive value (PPV), and F1 scores were determined.

**Results:**

A total of 2298 patients (59.5% female; median age 57 ± 15 years) recorded 86,816 ECGs between April 2019 and January 2021. The algorithm showed sensitivity of 0.956, specificity 0.985, PPV 0.996, NPV 0.847, and F1 score 0.976 for the detection of sinus rhythm. A total of 29 false-positive outcomes remained uncorrected within the same patients. The algorithm showed sensitivity of 0.989, specificity 0.953, PPV 0.835, NPV 0.997, and F1 score 0.906 for detection of atrial fibrillation. A total of 2 false-negative outcomes remained uncorrected.

**Conclusion:**

Our research showed high validity of the algorithm for the detection of both sinus rhythm and, to a lesser extent, atrial fibrillation. This finding suggests that the algorithm could function as a standalone instrument particularly for detection of sinus rhythm.

**Key Findings**

• High accuracy of sinus rhythm detection by the KardiaMobile (KM) algorithm is reported.
• The KM algorithm could function as a standalone algorithm for detection of sinus rhythm.
• In our study population, manual check for atrial fibrillation (AF) remained necessary because the KM algorithm showed a positive predictive value of 83.5%, which could lead to overdiagnosis of AF.

## Introduction

Atrial fibrillation (AF) is the most common cardiac arrhythmia, affecting approximately 3% of the world’s population.^1^^,^^2^ Because of its high prevalence, disease burden, and thrombotic risks, early detection and effective monitoring of AF are warranted. The gold standard for the diagnosis of AF is a physician’s assessment of the electrocardiogram (ECG).^1^^,^^3^ However, the diagnosis of AF can be challenging because of its possible paroxysmal or asymptomatic nature.^1^

Prolonged ambulatory ECG monitoring increases AF detection but is considered burdensome by many patients. New devices, such as wristbands, watches, and smartphones, provide the opportunity to perform ambulatory (single-lead) ECGs and could be used for extended monitoring with less burden.^4^^,^^5^ In addition to diagnostic purposes, these devices can be used for monitoring response to therapy and for self-management in patients with established AF.^5^^,^^6^ Patients using an accessible single-lead ECG monitoring device have report less arrhythmia-related anxiety and lower tendency to contact their physician.^7^ Ensuring the cost-effectiveness of telemonitoring programs requires the use of reliable medical devices embedded into clinical practice, where they replace, rather than add to, usual care.^8^ In addition, large-scale use is related to lower costs, which mostly rely on data interpretation independent of costly and scarce medical personnel. Relying on device algorithms for this verification requires high levels of certainty regarding the correct interpretation of registered data. The aim of this study was to evaluate the validity of a single-lead ECG device algorithm (KardiaMobile [KM], AliveCor, Mountain View, CA) in a real-life telemonitoring program (HartWacht, Cardiology Centers of the Netherlands, Amsterdam, The Netherlands) and its potential to function as a standalone instrument.

## Methods

We performed a retrospective analysis to evaluate the reliability of the KM algorithm as a standalone instrument for the interpretation of single-lead ECGs,^9^ specifically of sinus rhythm (SR) and AF, using all single-lead ECG data from the Dutch HartWacht program from April 2019 to January 2021.^10^

### Inclusion and exclusion criteria

Patients eligible for inclusion were enrolled in the telemonitoring program by their treating cardiologist. Inclusion criteria were patients aged 18 years or older, with complaints of palpitations of unknown origin, or diagnosed with paroxysmal persistent AF or a different supraventricular arrhythmia, or (near) collapse. Exclusion criteria for participation in the HartWacht program were unavailability of a smart device, being incapable of using a smart device, tremors, a pacemaker, or a known intermittent bundle branch block. Inclusion of patients was at the discretion of the cardiologist for diagnostic or follow-up purposes. Participation was voluntary, and patients were able to stop participation at any time.

### Telemonitoring program

All patients who were included in the HartWacht telemonitoring program received a KM device at their home address. Patients were requested to perform a single-lead ECG when they experienced palpitations of unknown origin, from a suspected arrhythmia, or from a previously diagnosed arrhythmia. To ensure the quality of these ECG recordings, patients were asked to view an online instruction video, complemented, if needed, with a personal consultation. Recordings were generated by placing 2 fingers from the left and right hands on the electrodes of the KM device. The captured ECG was transmitted to the patient’s smart device (eg, smartphone or tablet) through wireless communication and automatically interpreted by the ECG device algorithm (KM). The patient was then shown an assessment of the captured cardiac rhythm (eg, “normal rhythm” or “possible AF”).^11^ Automated uploading of the ECG recording to the patient’s electronic health record enabled manual checking of all incoming ECGs within 48 hours of reception by a dedicated telemonitoring team, consisting of specialized nurses supervised by a cardiologist with 24/7 availability. If ECG recordings were not eligible for assessment because of artifacts, patients were requested to perform a new recording.

### Interpretation by the KM algorithm and telemonitoring team

The ECG device algorithm KM classified cardiac rhythms into “normal”, “possible AF”, “tachycardia”, “bradycardia”, “unclassified”, “too short”, “unreadable”, and “no analysis”. All ECG recordings also were checked manually by the telemonitoring team. The team used several additional classifications, such as “atrioventricular block (AV block)”, “premature atrial contractions (PAC)”, “premature ventricular contractions (PVC)”, and “sinus arrest”. Atrial flutter was labeled as AF. ECGs that could not be classified using these labels were labeled as “other”, with subsequent interpretation in a free text bar.

ECGs that were classified by the telemonitoring team as “other” were excluded from this study, as were the ECG device algorithm labels “tachycardia”, “bradycardia”, “unclassified”, “too short”, “unreadable”, and “no analysis”, because ECGs with these labels would always require a manual double-check by medical personnel. For example, tachycardia could refer to sinus tachycardia but also could refer to a potentially life-threatening ventricular tachycardia requiring immediate action by a cardiologist. Therefore, only ECG device algorithm assessments “normal” and “possible AF” were included in this study.

### Comparison of interpretation by the device algorithm and telemonitoring team

ECG interpretation by the ECG device algorithm was compared to interpretation by the telemonitoring team to evaluate the diagnostic accuracy of the algorithm. The telemonitoring team consisted of trained eHealth nurses and supervising cardiologists and is considered the gold standard for ECG interpretation. If both assessments of an ECG classified the rhythm equally, the ECG was labeled as “true positive”. If the ECG device algorithm labeled the ECG differently from the telemonitoring team, the ECG was labeled as “false positive” or “false negative”. An overview is given in Tables 1 and 2.

**Table 1:** Determination of diagnostic accuracy for “possible AF” assessments by the ECG device algorithm vs the telemonitoring team

Possible AF∗		Telemonitoring team	
		Positive	Negative
	Positive	**True positive**	**False positive**
		Device algorithm: possible AF	Device algorithm: possible AF
		Telemonitoring team: AF	Telemonitoring team: all rhythms except AF
Device algorithm			
	Negative	**False negative**	**True negative**
		Device algorithm: SR	Device algorithm: SR
		Telemonitoring team: AF∗	Telemonitoring team: all rhythms except AF

AF = atrial fibrillation; ECG = electrocardiogram; SR = sinus rhythm.

∗ AF defined as atrial fibrillation or atrial flutter.

**Table 2:** Determination of the diagnostic accuracy for “SR” assessments by the ECG device algorithm vs the telemonitoring team

SR∗ (with ectopy)		Telemonitoring team	
		Positive	Negative
	Positive	**True positive**	**False positive**
		Device algorithm: SR	Device algorithm: SR
		Telemonitoring team: SR	Telemonitoring team: all rhythms except from SR
Device algorithm			
	Negative	**False negative**	**True negative**
		Device algorithm: possible AF	Device algorithm: possible AF
		Telemonitoring team: SR	Telemonitoring team: all rhythms except from SR

Abbreviations as in Table 1.

∗ SR with or without ectopy (premature atrial contraction, premature ventricular contraction, atrioventricular block type 1).

All “false-negative” (AF) and “false-positive” (SR) ECGs were manually checked to determine the clinical relevance of the potentially missed diagnosis. For ECGs with a “false negative” for detection of AF (non-AF diagnosed by the ECG device algorithm; AF diagnosed by the telemonitoring team), all prior and subsequent ECGs were checked for AF to evaluate whether AF had remained undiagnosed by the ECG device algorithm. If AF was registered in a prior or subsequent ECG with a true positive outcome, the false-negative outcome was considered clinically irrelevant. The same logic applied to SR. If false-positive outcomes (SR diagnosed by the ECG device algorithm; an arrhythmia diagnosed by the telemonitoring team) were corrected based on prior or subsequent arrhythmia recordings, the false-positive ECG was considered clinically irrelevant.

Two cardiologists (MW, GAS) assessed a random selection of ECGs to evaluate bias by the lack of blinding of the telemonitoring team in their interpretations of the recording.^12^

This study was performed in accordance with the Declaration of Helsinki as revised in 2013. All data were analyzed at Cardiology Centers of the Netherlands in accordance with its privacy statement.

### Statistical analysis

SPSS Version 27 (SPSS Inc, Chicago, IL) was used to perform further analyses. Data on sex and date of birth were gathered to determine baseline characteristics. Assessments by both the KM algorithm and the telemonitoring team were collected for further analysis to support/reject our hypothesis. Continuous variables (eg, age) are given as mean ± SD or, if not normally distributed, as median [interquartile range]. Other variables are given as frequency (percentage).

By using assessments of the telemonitoring team as a reference standard, we determined the sensitivity, specificity, positive predictive value (PPV), negative predictive value (NPV), and F1 scores of the KM algorithm. Cohen’s kappa was used to determine the inter-rater reliability between the nonblinded telemonitoring team and the blinded cardiologists. We interpreted the results according to Cohen’s Kappa definition as follows: 0.0–0.20 none agreement; 0.21–0.39 minimal agreement; 0.40–0.59 weak agreement; 0.60–0.79 moderate agreement; 0.80–0.90 strong agreement; and >0.90 almost perfect agreement.^13^

## Results

**Figure 1:**
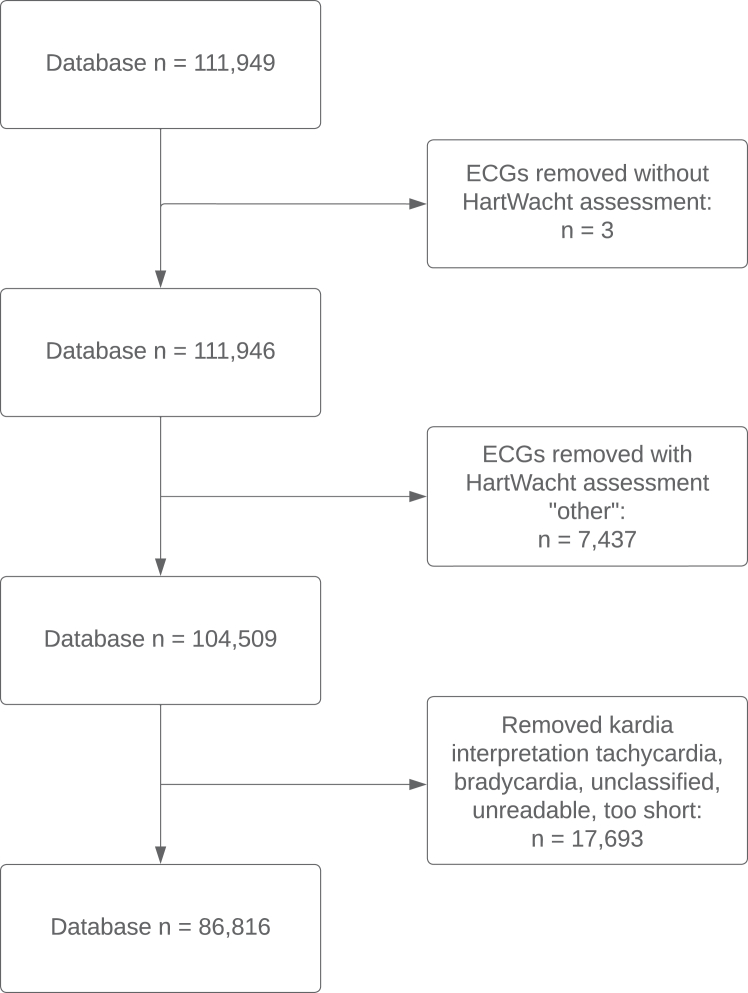
Flowchart exclusion database. ECG = electrocardiogram.

The database consisted of 111,949 recordings for analysis. Three ECGs were omitted because of missing assessment by the telemonitoring team, 7437 ECGs (6%) were excluded from the analysis because they were classified as “other” by the telemonitoring team, and 17,693 ECGs (15%) were excluded because they were classified as tachycardia, bradycardia, unclassified, unreadable, or too short by the ECG device algorithm. The remaining 86,816 ECG recordings, recorded by 2298 patients (median age 60 [47–69] years; 1367 female [59.5%]), were included in the final analysis (Table 3 and Figure 1) and were classified by the ECG device algorithm as possible AF (20,030 ECGs [23.1%]) or normal (66,786 ECGs [76.9%]). The telemonitoring team classified 69,585 ECGs (80%) as SR (with or without ectopy) and 16,905 ECGs (19%) as AF (with or without flutter). A total of 234 ECGs (<1%) were classified as other arrhythmias, such as second-degree AV block, and 92 ECGs (<1%) were classified as unreadable.

**Table 3:** Baseline patient characteristics

	Total (n = 86,816)	
	n	%
Patients	2298	100
Sex		
Male	931	40.5
Female	1367	59.5
KM assessment		
SR	66,786	77
Possible AF	20,030	23

	IQR	Median	Range
Age (y)	47–69	60	1691

IQR = interquartile range; KM = KardiaMobile; other abbreviations as in Table 1.

### Comparison of classification by the ECG device algorithm and telemonitoring team

**Figure 2:**
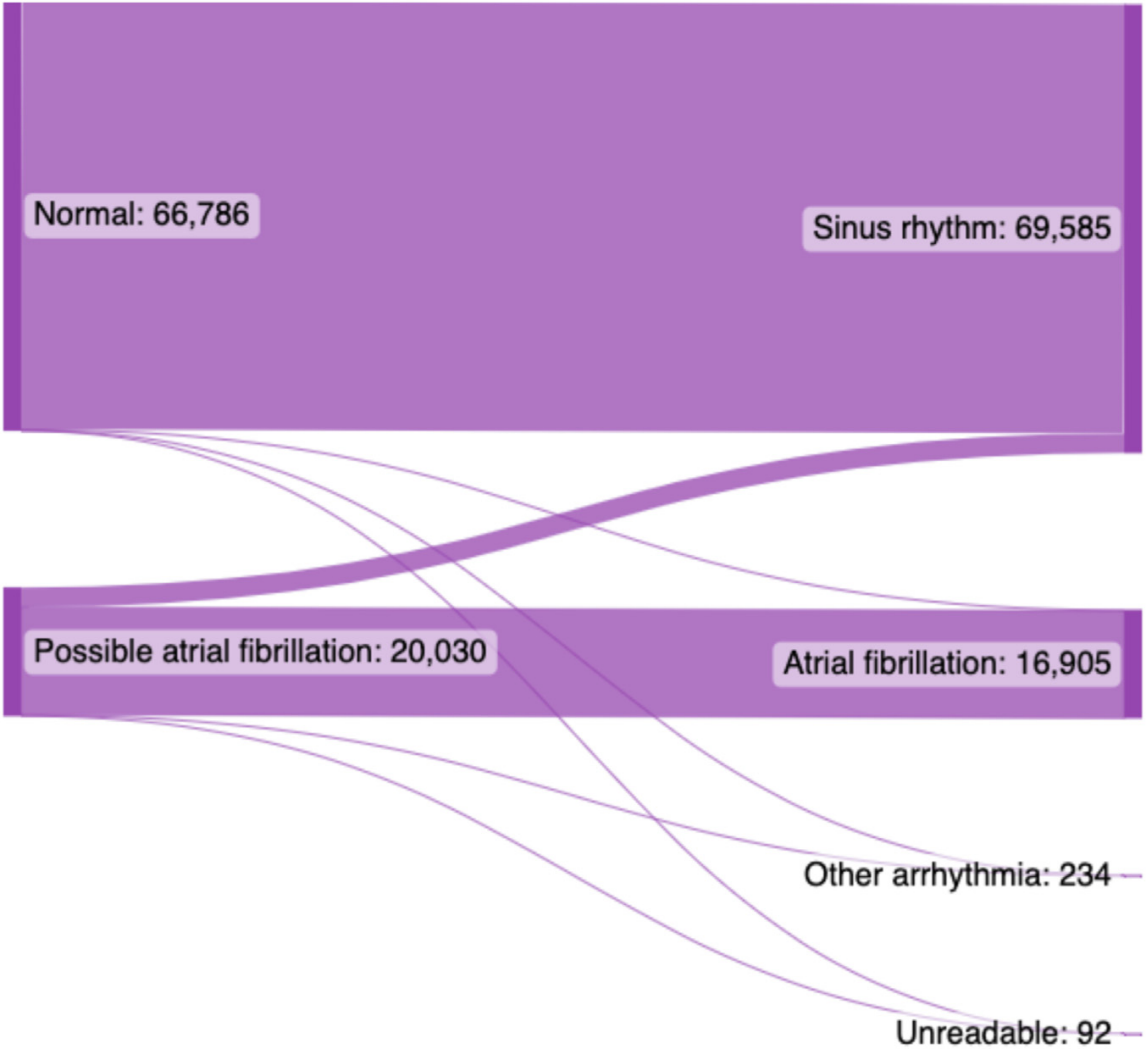
Interpretation of electrocardiograms (ECGs) by the ECG device algorithm **(left)** and by the telemonitoring team **(right).**

For SR, there was 99.6% agreement between the interpretation by the ECG device algorithm and the telemonitoring team (66,786 ECGs vs 66,520 ECGs, respectively). A total of 266 ECGs were classified by the ECG device algorithm as SR, and by the telemonitoring team as AF (150 ECGs [<1%]), atrial flutter (37 ECGs [<1%]), other arrhythmias (62 ECGs [<1%]), and unreadable (17 ECGs [<1%]) (Table 4 and Figure 2).

**Table 4:** Interpretation of ECGs by ECG device algorithm and the telemonitoring team

	SR (ECG device algorithm) (n = 66,786)		Possible AF (ECG device algorithm) (n = 20,030)	
	n	%	n	%
SR	66,520	99.6	3065	15
Without ectopy	60,453	90.5	675	3
With ectopy	6067	9.1	2390	12
AF	150	<1	16,692	83
Atrial flutter	37	<1	26	<1
Other arrhythmias	62	<1	172	<1
Malfunction or unreadable	17	<1	75	<1

Abbreviations as in Table 1.

These 266 ECGs (<1%) were recorded by 104 patients (4.5%). In 83 patients, prior or subsequent ECG recordings demonstrated and correctly classified the arrhythmias and therefore were considered clinically irrelevant misinterpretations. The remaining 21 patients recorded a total of 29 false-positive ECGs without having another recording with arrhythmias. Therefore, the ECG device algorithm for SR showed sensitivity of 0.956, specificity 0.985, PPV 0.996, NPV 0.847, and F1 score 0.976 (Table 5).

**Table 5:** Diagnostic accuracy of “SR” and “possible AF” classifications by the ECG device algorithm

Sinus rhythm		Telemonitoring team		
		Positive	Negative	
ECG device algorithm	Positive	66,520	266	PPV 99.6%
	Negative	3065	16,965	NPV 84.7%
		Sensitivity 95.6%	Specificity 98.5%	

Possible AF		Telemonitoring team		
		Positive	Negative	
ECG device algorithm	Positive	16,718	3312	PPV 83.5%
	Negative	187	66,599	NPV 99.7%
		Sensitivity 98.9%	Specificity 95.3%	

NPV = negative predictive value; PPV = positive predictive value; other abbreviations as in Table 1.

For AF, there was 83.5% agreement between the ECG device algorithm and the telemonitoring team (20,030 ECGs vs 16,718 ECGs, respectively). The remaining 3312 ECGs were classified by the telemonitoring team as SR (3065 ECGs [15.3%]), other arrhythmias (172 ECGs [<1%]), and unreadable (75 ECGs [<1%]) (Table 4 and Figure 2).

The ECG device algorithm misclassified AF as SR in 187 ECGs (<1%). These 187 ECGs were recorded by 68 patients (3%). In 66 patients, prior or subsequent ECG recordings demonstrated and correctly classified AF and therefore were considered clinically irrelevant misinterpretation. The remaining 2 patients recorded a total of 2 false-negative ECGs. The ECG device algorithm for AF showed sensitivity of 0.989, specificity 0.953, PPV 0.835, NPV 0.997, and F1 score 0.906 (Table 5).

The level of agreement (Cohen’s kappa) between cardiologists and the telemonitoring team for the determination of SR was 0.88 and 0.90, respectively, and of AF was 0.88 and 0.90, respectively, which corresponds to strong reliability of the “nonblinded” HartWacht team.

## Discussion

We evaluated the potential of the ECG device algorithm (KM) to replace manual interpretations of ECGs in a real-life telemonitoring setting (HartWacht) using a large representative dataset of more than 2200 unique patients with more than 86,000 ECGs. We conclude that standalone algorithm interpretation of SR ECGs is feasible and that manual check of these ECGs might be omitted. Omitting the double-check of SR ECGs reduces the number of ECGs that need to be checked by 64% and therefore leads to a significant improvement in cost-effectiveness. Whether the algorithm for AF still requires double-checking, despite its high sensitivity and specificity, remains unclear. The PPV of the algorithm is lower (83.5%) for the detection of AF, which indicates that in this population, with a high prevalence of arrhythmias, the algorithm overdiagnosed AF. Thus, unsupervised use of the algorithm for AF might lead to treatment without a solid indication.

The potential of the ECG device algorithm to replace manual ECG assessment in SR is a result of its high diagnostic accuracy. Previous studies on this topic showed heterogeneous results on the diagnostic accuracy of the ECG device algorithm. A systematic review comparing multiple studies using the KM algorithm showed sensitivity for AF detection ranging between 67% and 100%.^4^ This large difference in reported sensitivity could be due to the inclusion of unclassifiable ECGs, as studies that excluded these ECGs reported higher sensitivity. The majority of studies determined the accuracy of AF detection using the KM device, without commenting on the accuracy of SR detection. Therefore, comparing previous studies to our results is not possible. Moreover, all studies included in the review used a 12-lead ECG as the reference standard, which is the current gold standard. Our results are based on interpretation by the KM algorithm and the telemonitoring team of the same single-lead ECG, with no 12-lead ECG available for cross-validation. We did not aim to cross-check with a 12-lead ECG because the HartWacht telemonitoring program is specifically designed to interpret a single-lead ECG. However, a study that compares a single-lead ECG to a 12-lead ECG (interpreted by 2 electrophysiologists) has shown sensitivity of 100% and specificity of 94%, which suggests high similarity between the results of single-lead vs 12-lead ECGs.^14^

Other studies have evaluated ECG interpretation by an algorithm vs a dedicated team with study designs similar to ours. Chan et al^15^ performed a prospective screening study for AF detection in a population at high risk for ischemic stroke, using both KM single-lead ECGs and photoplethysmography, with a yield of 28 AF diagnoses in 1013 patients. All incoming data were double-checked by 2 cardiologists, resulting in moderate sensitivity (71%) but high specificity (99%) when using the ECG device algorithm.^15^ Another study found similar sensitivity (75%) and specificity (98%) when using the KM algorithm as an AF screening tool in 10,735 elderly patients.^16^ Interestingly, we demonstrated lower but still high specificity rates (95%) compared to these studies, which could be due to discrepancies in the target population. One of these studies targeted the “healthy” elderly population, whereas the other study focused on patients with risk factors such as diabetes, hypertension, and older age. These differences in prevalence of AF could affect the diagnostic accuracy of the KM device. HartWacht only included patients with a high suspicion of arrhythmias, which could explain the differences in diagnostic accuracy.^17^

Most studies concentrated on AF detection in high-risk patients. However, our study used real-life data for monitoring and detecting several cardiac arrhythmias in a large patient population for which the determination of SR is equally important for reassurance purposes in case of complaints that are not caused by potentially harmful cardiac arrhythmias. This approach led to 64% of incoming ECGs demonstrating SR. The potential to omit manually double-checking of ECGs showing SR would significantly decrease workload and enable the cost-effective scale-up of any telemonitoring system. As stated, we found that ECGs showing SR did not require double-checking, as the PPV of the algorithm was almost 100%. Because the accuracy of SR classification has not been assessed previously, comparison to our results is challenging. However, in other disciplines such as radiology, use of image interpretation by algorithms instead of physicians is more widespread. For example, interpreting mammograms is largely performed by algorithms.^18^

In general, disagreement ≤1% between the algorithm and the physician is considered acceptable because this disagreement in some cases means that the algorithm outperforms the radiologist.^19^ Applying the same logic to algorithmic classification of eHealth programs for cardiovascular disease implies great potential for upscaling.

### Study limitations

We used a large-scale, real-world dataset of patients who are actively included in the HartWacht eHealth system, resulting in a large number of ECGs from patients with a large variety of cardiac rhythms. All ECGs were systematically double-checked by a dedicated team of specialized nurses under the supervision of a cardiologist. This team was not blinded to the ECG device algorithm’s rhythm classification, which may have caused observer bias. To discard this bias, we showed similarly high agreement between the team and the blinded cardiologists.

In our study, single-lead ECGs were not cross-validated with the gold standard 12-lead ECG because the goal of our study was not to validate the ECG device algorithm but to determine the safety of omitting manual double-checking of ECGs. The fact that the results of our study were similar to those of the telemonitoring team that performed a 12-lead reference check adds to the reliability of our results.

In addition, because of incomplete baseline patient characteristics we were unable to accurately verify the prevalence of arrhythmias in the study population. This may have implications for the outcomes of this study that should be taken into account when interpreting the results.

## Conclusion

Standalone algorithm interpretation of SR ECGs by the KM algorithm, without additional manual checking, is feasible. The ECG device algorithm used during the study period did not allow for standalone AF detection. Omitting the manual check of SR ECGs significantly reduces workload and facilitates scaling-up of existing eHealth infrastructures.
